# Epilepsy phenotype in individuals with chromosomal duplication encompassing *FGF12*

**DOI:** 10.1002/epi4.12396

**Published:** 2020-05-09

**Authors:** Marjolein H. Willemsen, Himanshu Goel, Judith S. Verhoeven, Hilde M. H. Braakman, Nicole de Leeuw, Alison Freeth, Berge A. Minassian

**Affiliations:** ^1^ Department of Clinical Genetics Maastricht University Medical Centre Maastricht The Netherlands; ^2^ Department of Human Genetics Radboud University Medical Center Nijmegen The Netherlands; ^3^ Donders Institute for Brain Cognition and Behaviour Radboud University Nijmegen The Netherlands; ^4^ Hunter Genetics Waratah NSW Australia; ^5^ University of Newcastle Callaghan NSW Australia; ^6^ Department of Neurology Academic Center for Epileptology Kempenhaeghe and Maastricht UMC+ Heeze The Netherlands; ^7^ Department of Pediatric Neurology Amalia Children's Hospital Radboud University Medical Center Nijmegen The Netherlands; ^8^ Program in Genetics and Genome Biology Hospital for Sick Children Research Institute Toronto ON Canada; ^9^ Institute of Medical Science University of Toronto Toronto ON Canada; ^10^ Division of Neurology Department of Pediatrics University of Texas Southwestern Dallas TX USA

**Keywords:** developmental regression, epilepsy, *FGF12*, intellectual disability, LINE, microduplication 3q28q29

## Abstract

Intragenic mutations in *FGF12* are associated with intractable seizures, developmental regression, intellectual disability, ataxia, hypotonia, and feeding difficulties. *FGF12* duplications are rarely reported, but it was suggested that those might have a similar gain‐of‐function effect and lead to a more or less comparable phenotype. A favorable response to the sodium blocker phenytoin was reported in several cases, both in patients with an intragenic mutation and in patients with a duplication of *FGF12*. We report three individuals from two families with *FGF12* duplications. The duplications are flanked and probably mediated by two long interspersed nuclear elements (LINEs). The duplication cases show phenotypic overlap with the cases with intragenic mutations. Though the onset of epilepsy might be later, after the onset of seizures both groups show developmental stagnation and regression in several cases. This illustrates and further confirms that chromosomal *FGF12* duplications and intragenic gain‐of‐function mutations yield overlapping phenotypes.

## 1 INTRODUCTION

Mutations in *fibroblast growth factor 12 (FGF12)*, formerly known as *fibroblast growth factor homologous factor 1 (FHF1),* are associated with an early infantile epileptic encephalopathy 47 (MIM 617 66).^1^, ^2^, ^3^, ^4^, ^5^ The phenotype, including moderate to mostly severe developmental delay (DD)/intellectual disability (ID), early‐onset drug‐resistant epilepsy with frequent status epilepticus, additional neurological symptoms such as cerebellar ataxia, hypotonia, and feeding difficulties, is attributed to a toxic gain of function of *FGF12,* assumed to lead to increased neuronal excitability.^3^ *FGF12* encodes FGF12/FHF1, one of a family of four proteins that interact with C‐termini, and modulates activities of voltage‐gated sodium channels (Na_v_).^3^ Mutations in voltage‐gated sodium channels are well‐known causes of epilepsy and epileptic encephalopathy.

Nineteen intragenic mutations in *FGF12* have been reported, including recurrent mutations p.Arg52His and p.Arg114His.^1^, ^2^, ^3^, ^4^, ^5^, ^6^ Only one patient with a small chromosomal duplication involving *FGF12* has been reported, who showed similarities with the phenotype in patients with an intragenic mutation, including drug‐resistant epilepsy, severe DD/ID, cerebellar ataxia, and feeding difficulties.^7^, ^8^ A favorable response to the sodium blocker phenytoin was reported in several cases, including this chromosomal duplication case.^3^, ^7^

We report three additional individuals from two families with a (partial) duplication of *FGF12* to further confirm the epilepsy/ID phenotype and to further compare this with the intragenic mutation cases.

## 2 CLINICAL FINDINGS

Individuals were ascertained during routine clinical diagnostics for their intellectual disability and epilepsy at the departments of human genetics of the Radboudumc/MUMC+ in the Netherlands (individual 1) and the Department of Hunter genetics, Waratah, Australia (individuals 2 and 3). Clinical features are summarized in Table 1.

**Table 1:** Clinical features of individuals with chromosomal duplication encompassing *FGF12*

	Individual 1	Individual 2	Individual 3
Genome coordinates *FGF12* duplication (chr3; hg19)	191 860 089‐192 451 114	191 876 968‐192 454 685	191 876 968‐192 454 685
Age at last examination	10 y	3 y	30 y
Age at seizure onset	12 mo	13 mo	1 mo
Seizure types	Tonic‐clonic, atonic, tonic, myoclonic, autonomic	Generalized tonic‐clonic and history of febrile seizures Myoclonic jerks	Generalized tonic‐clonic
EEG	Sowed background and multifocal seizures	Normal	Normal
AED treatment	Sodium valproate, clobazam, lamotrigine, and topiramate	Valproate	Valproate, carbamazepine, lamotrigine, levetiracetam
Other treatments	Vagal nerve stimulator Ketogenic diet	—	—
DD/ID	Severe DD/ID No speech Wheelchair dependent	Developmental delay	Developmental delay
Other neurological symptoms	Ataxia	Unsteady	Unsteady and migraine
Brain MRI	Bilateral delayed myelination in the parieto‐occipital region	Mild prominence of the subarachnoid space in the frontal regions bilaterally otherwise normal	Normal
Feeding difficulties	+, progressive, requiring PEG	No	No
Recurrent infection	+, upper airway	No	Perianal abscess Dental infections

### 2.1 Individual 1

A 10‐year‐old boy presented with severe DD/ID, absent speech, wheelchair dependency, and drug‐resistant epilepsy. Afebrile, nocturnal tonic‐clonic seizures started at the age of 12 months. At the age of three years, he presented with seizures that were qualified as autonomic seizures. In the prephase, there was a tachycardia as autonomic phenomenon, followed by a tonic seizure with prominent hypersalivation, and retching or vomiting. He also had apneas, focal seizures, and myoclonic and atonic seizures. Antiepileptic drug regimen included sodium valproate, clobazam, lamotrigine, and topiramate. In addition, he followed a ketogenic diet from four to ten years of age, and he received a vagus nerve stimulator at the age of six years. Electroencephalography (EEG) showed slowed background activity and multifocal epileptic discharges (age three years). Seizure frequency varied, with alternating periods of poor seizure control and periods with lower seizure frequency. In his first years of life, seizures were clustering with clusters of 1 to 2 tonic‐clonic insults every day during 5‐6 days, followed by a seizure‐free week. After that period, he had a mean seizure frequency of 3‐4 seizures per week. When growing older, he had periods of up to 6‐8 weeks with very few seizures (only tonic seizures, no tonic‐clonic or autonomic seizures), often followed by several weeks of daily seizures. Brain MRI showed delayed myelination in the parieto‐occipital regions bilaterally.

Since the age of 18 months, speech‐language delay was obvious and he presented with ataxia. Since the age of 3 years, a general deterioration was observed and he developed progressive feeding difficulties for which tube feeding (percutaneous endoscopic gastrostomy (PEG)) was required. He had frequent upper airway infections, reflux, and constipation.

### 2.2 Individuals 2 and 3

Individual 2 is a four‐year‐old boy with global DD, epilepsy, and dysmorphic facial features (ie, bilateral epicanthus, down‐slanting palpebral fissures, hypotelorism, metopic ridge, flat nasal bridge, midface hypoplasia, thin upper lip, smooth philtrum, and low muscle tone). He was born at 35 weeks’ gestation after a pregnancy complicated by hypertension, low vitamin D, and uncontrolled epilepsy. His mother took valproate (stopped at 4‐5 weeks’ gestational age), carbamazepine, and lamotrigine. He had a low birthweight of 2175 g. He had a febrile convulsion at 13 months of age associated with bronchiolitis and pneumonia. An EEG during wakefulness at this time was normal.

At 15 months of age, he presented with a cluster of brief tonic‐clonic seizures and he started sodium valproate treatment. An EEG at the age of two demonstrated generalized epileptic activity. Brain MRI was within normal limits, with mild prominence of subarachnoid space in the frontal regions bilaterally. By four years of age, he continued to have predominantly clusters of generalized tonic‐clonic seizures while treated with sodium valproate. At his last visit, he was 5 years old and was able to walk independently, although he had an uncoordinated gait. He was drooling, but could speak clearly; however, his vocabulary was significantly reduced. He has been diagnosed with autism spectrum disorder level 3 (requiring very substantial support). His developmental delay was moderate, with an onset from early childhood. There was no regression. The mother of patient 2 has ID and epilepsy as well (see below). His father does not have ID or epilepsy.

Individual 3 is the 31‐year‐old mother of individual 2. She was diagnosed with epilepsy at the age of one month. She presented with generalized tonic‐clonic seizures. She used many antiepileptic drugs and is currently on sodium valproate and carbamazepine. With this medication, she was still having regular breakthrough seizures. She had special education in high school and has speech problems. She had a mild‐to‐moderate ID, with an onset of developmental delay from early childhood, and limited reading and writing abilities. There was no regression reported. She did not work and required a care supervisor for her daily activities. Because of her disabilities, she was not involved in care of her son (patient 2). In addition, she reported unsteadiness on her feet frequently. She had no significant facial dysmorphic features. Her brain CT was normal. Her parents and siblings have no learning problems or seizures and no chromosomal abnormalities.

## 3 GENETIC ANALYSIS

**Figure 1:**
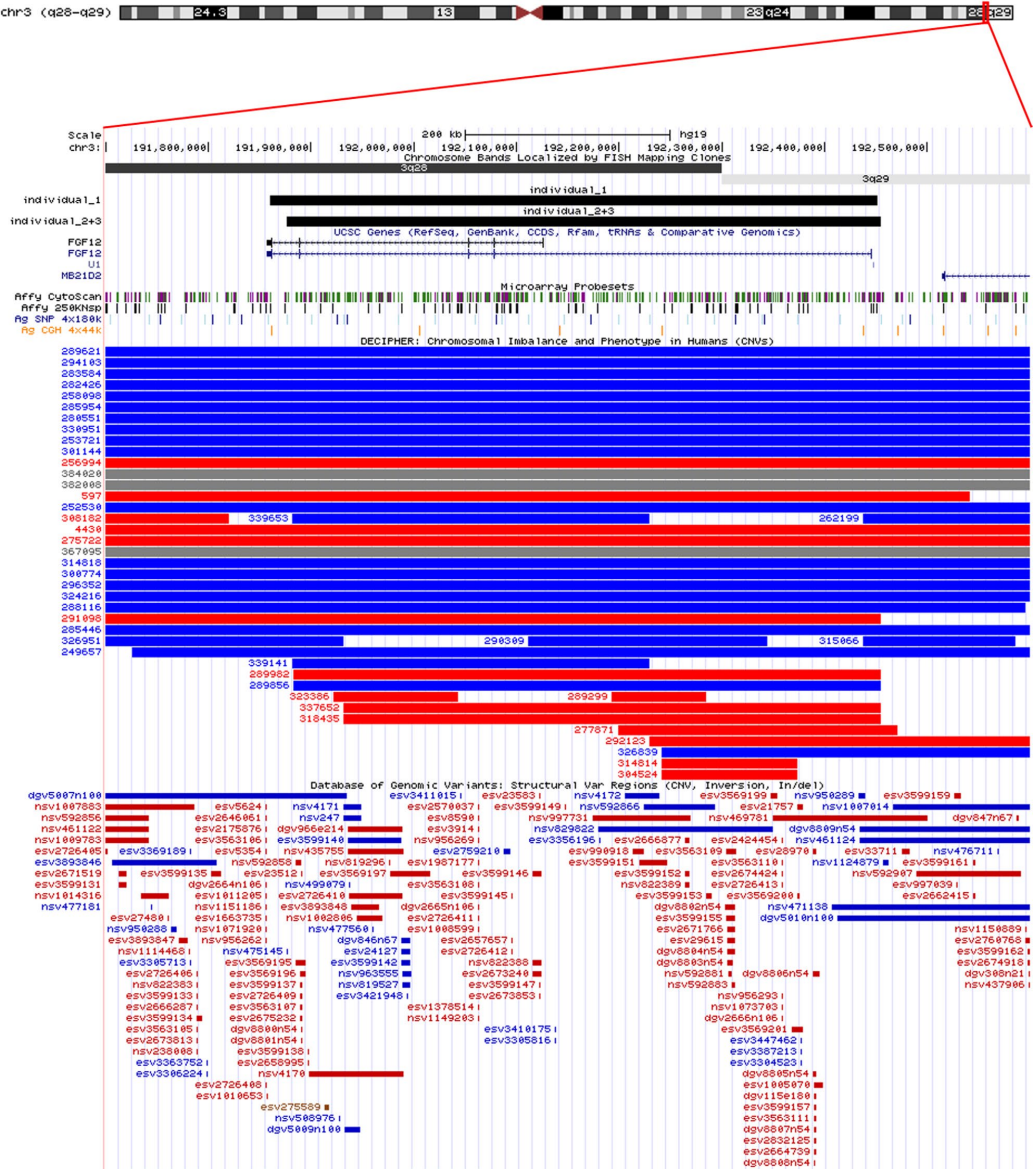
Graphic presentation of the overlapping gains in band q28q29 of chromosome 3, encompassing FGF12. Schematic representation of chromosome 3 with the 3q28q29 region enlarged in the lower part of the figure (screen shot of the UCSC genome browser build 37/hg19 (http://genome.ucsc.edu/)) with gains detected in individuals 1, 2, and 3 shown as black solid rectangles above the genes in this region. The small vertical bars indicate the probe coverage of the different array platforms, and the blue and red rectangles represent gains and losses, respectively, in the DECIPHER database and the Database of Genomic Variants^10^

In individual 1, chromosomal microarray was performed with the Affymetrix CytoScan HD platform and revealed a de novo 590 kb copy number gain in chromosomal region 3q28q29 (191 860 089‐192 451 114, human genome build hg19) encompassing exclusively *FGF12*. The gain fully encompasses *FGF12* with the proximal breakpoint being located in the noncoding last exon or slightly downstream (see Figure 1). He had normal DNA testing for fragile X syndrome, *MECP2*, Angelman syndrome, POLG, mitochondrial DNA, and whole‐exome sequencing.

In individuals 2 and 3, microarray was done using the Agilent SurePrint G3'' CGH + SNP Microarray 4x180k platform. This revealed three copy number variants in individual 2. Two were maternally derived, a gain in 3q28q29 (0.58 Mb, 191 876 978‐192 454 675 (hg19)) and a 8q21.12 deletion (0.20 Mb, 78 521 326‐78 726 223 (hg19)), and the other is a paternally derived 4q26 deletion (0.13 Mb, Mb positions (hg19):114 876 387‐115 005 411). Fragile X testing was normal. In individual 3, two copy number variants were identified: the same gain in 3q28q29 and 8q21.12 deletion that were identified in her son.

The overlapping gains in band q28q29 of chromosome 3 of individuals 1, 2, and 3 are shown in Figure 1.

Whole‐exome sequencing was not done in individuals 2 and 3.

## 4 DISCUSSION

We noted several similarities in disease course between our *FGF12* duplication cases and the previously reported case by Shi et al in the form of stagnation in development, refractory seizures, onset of additional neurological features including ataxia or unsteady/clumsy gait, and onset of feeding difficulties.^7^ In addition, we noticed an important overlap in epilepsy and neurodevelopmental phenotype between the few patients with *FGF12* duplications and previously reported patients with intragenic, presumably gain‐of‐function mutations in *FGF12*, though there may be differences in severity of the ID and epilepsy burden; for example, the phenotypes of individuals 2 and 3 seem to have a less severe presentation with respect to intellectual functioning and epilepsy burden. However, also in the group of intragenic mutations severity and presentation can vary slightly. In both groups, seizure types and presentations are multiform including generalized seizures with different presentations as well as focal seizures in some patients.^1^, ^2^, ^3^, ^4^, ^5^ In both groups, seizures are often drug‐resistant. In some patients, a good response to phenytoin was observed.^1^, ^2^, ^4^, ^6^, ^7^ In the present patients, phenytoin was not tried. In individual 1, other sodium channel blockers (oxcarbazepine and lamotrigine) did not result in seizure control. In individuals 2 and 3, there was reasonably good seizure control with sodium valproate. In both groups, cerebral MRI scans show mostly normal results or mild nonspecific findings. All but one patient were reported to have variable neurodevelopmental delay.^1^, ^2^, ^3^, ^4^, ^5^, ^6^, ^7^ Individuals with later seizure onset, either with a duplication or a mutation, show a clear developmental stagnation/regression after seizure onset. Ataxia and feeding difficulties were present in a subset of the cases of both groups.^1^, ^3^, ^7^ Based on the current and previous cases in both groups, there is no consistent facial phenotype recognizable.

A gain‐of‐function effect on sodium channel gating was proven for the Arg114His and Arg52His mutations.^3^ We hypothesize that the duplications encompassing *FGF12* may have a similar gain‐of‐function effect on sodium channel gating. The duplications of the present patients and the patient reported by Shi et al are flanked and probably mediated by two long interspersed nuclear elements (LINEs) (L1P2A‐A and L1P2A‐B), as shown by Shi et al^7^ In the DECIPHER database (http://decipher.sanger.ac.uk), three additional duplication cases exclusively encompassing *FGF12* are reported without information about the phenotype. In addition, more than 20 additional (mostly) much larger gains are reported. In several cases, phenotypic information is lacking, and in other cases, it may not be complete. In some cases, a phenotype with ID and/or EEG abnormalities is mentioned. However, these gains encompass additional genes and are therefore difficult to compare with the present duplication in which *FGF12* is involved exclusively. Recently, a complex chromosomal rearrangement, including a much larger duplication encompassing the whole *FGF12* gene and an inv dup del(9p), was reported in a patient with West syndrome.^9^ The authors suggested a relation with the *FGF12* duplication and the epilepsy phenotype. However, it is difficult to compare this case with our cases because of the complex chromosome rearrangement. In addition, several deletions overlapping with this recurrent copy number variant region are reported in DECIPHER and our in‐house database. In some of these patients, (mild) ID and/or autism spectrum disorder were reported, suggesting that dosage of *FGF12* is important for neurodevelopment. Epilepsy is not reported in the deletion cases. Although the breakpoints of the duplications and deletions in these patients seem to differ a little bit, this may be in fact mostly due to differences in array platforms used: 44k, 180k, 250k, and CSHD (2.7 million), listed with increasing resolution.

Individual 2 has two additional copy number variations. The paternally inherited chromosome 4q26 deletion results in a partial deletion of *Arylsulfatase J (ARSJ*), with unknown disease association, and high expression in fetal kidneys. Related to the presented phenotype, it is unlikely to be clinically relevant. The maternally inherited chromosome 8q21.12 deletion does not involve any gene and is not likely related to the phenotype.

We observed that chromosomal *FGF12* duplications and intragenic gain‐of‐function mutations yield overlapping phenotypes. Though the onset of epilepsy might be later, it may be followed by developmental stagnation and regression in both groups. Identification of additional chromosomal *FGF12* duplication cases should further delineate the clinical spectrum and overlap with the intragenic mutations. In addition, functional studies may support the expected underlying gain‐of‐function effect on sodium channel gating for the duplication cases as well.

## CONFLICT OF INTEREST

None of the authors has any conflict of interest to disclose. We confirm that we have read the journal's position on issues involved in ethical publication and affirm that this report is consistent with those guidelines.
